# Editorial: Role of CD1- and MR1-Restricted T Cells in Immunity and Disease

**DOI:** 10.3389/fimmu.2019.01837

**Published:** 2019-08-06

**Authors:** Kazuya Iwabuchi, Luc Van Kaer

**Affiliations:** ^1^Department of Immunology, Kitasato University School of Medicine, Sagamihara, Japan; ^2^Department of Pathology, Microbiology and Immunology, Vanderbilt University School of Medicine, Nashville, TN, United States

**Keywords:** CD1, MR1, natural killer T cells, mucosal-associated invariant T cells, non-peptide antigens, immunity, immune-mediated disease

Products encoded by the major histocompatibility complex (MHC) present peptide antigens to T lymphocytes. MHC class I and class II products present peptide antigens to CD8^+^ or CD4^+^ T lymphocytes, respectively. In addition to these classical MHC class I molecules (also known as MHC class Ia molecules), numerous non-classical MHC class I molecules (also known as MHC class Ib molecules) have been identified (1). Some of these products are encoded by genes within the MHC, whereas others are encoded by genes outside the MHC. Key differences between class Ia and class Ib molecules are that the former are highly polymorphic, are expressed by most cell types, and present pathogen-derived peptides to CD8^+^ T cells, whereas the latter are oligomorphic, are less widely expressed, and are diverse in their capacity to present antigens to subsets of T lymphocytes with innate or adaptive immune functions. Some class Ib molecules such as mouse Qa-1 (HLA-E in human) and H-2M3 bind peptide antigens, others such as CD1 (cluster of differentiation-1) and MR1 (MHC-related protein-1) bind non-peptide antigens, and again others such as the mouse TL (thymus leukemia) and the human MIC (MHC class I polypeptide-related sequence) antigens are not known to bind antigen of any kind. This Research Topic focuses on the immunological functions of T lymphocytes restricted by CD1 or MR1 molecules, which present lipids or small metabolites, respectively, to subsets of T lymphocytes with conventional or unconventional functions [see (2–5) for a general review of these topics].

The CD1 family of antigen-presenting molecules has been divided into two groups: Group 1 contains CD1a, CD1b, and CD1c, and Group 2 contains CD1d (6). Additionally, human CD1e is expressed intracellularly and is involved in the loading of lipid antigens onto the other CD1 isotypes, which are all displayed at the cell surface. Humans express both Groups 1 and 2 CD1 proteins, whereas mice only express Group 2 CD1 proteins (i.e., CD1d). Group 1 CD1 proteins present lipid antigens to T cells that generally express diverse T cell receptors (TCRs) and exhibit adaptive-like functions, whereas CD1d presents glycolipid antigens to subsets of T cells that express restricted TCRs and exhibit innate-like functions (7, 8). CD1-restricted T cells have been implicated in a wide variety of diseases, including cancer, infections, and autoimmune, inflammatory and metabolic diseases.

Seminal studies with human autoreactive T cell lines led to the identification of lipid-reactive T cells restricted by CD1 molecules (9). Subsequent studies identified CD1-restricted T cells specific for microbial lipids, especially those derived from the cell wall of mycobacterial species (10, 11). While some of the initial T cell clones isolated expressed γδ TCRs, it is now clear that the majority of CD1-reactive T cells express αβ TCRs. The antigen-binding groove of CD1 is hydrophobic and differs in size between distinct CD1 isoforms, which permits these molecules to bind diverse lipid or glycolipid antigens (7, 12). Some lipids, such as the self-antigen sulfatide, can bind with all CD1 isoforms. Each of the CD1 isoforms can present mycobacterial lipid antigens, albeit distinct in structure, to CD1-restricted T cells. Tetrameric CD1 molecules loaded with lipid antigens have been generated for all CD1 isotypes and have greatly assisted in understanding the biology of CD1-restricted T cells (5).

Most Group 1 CD1-restricted T cell clones described are autoreactive, and likely represent a substantial part of the T cell repertoire in humans, estimated at 1/300 to 1/10 circulating T cells (13). The majority of these T cell clones express diverse αβ TCRs, although subsets of Group 1 CD1-restricted T cells expressing conserved TCRs have also been identified (14). As argued in the review article by Bagchi et al., emerging evidence indicates that Group 1 CD1-restricted T cells can contribute to autoimmunity, especially in diseases associated with dyslipidemia. For example, CD1c-restricted T cells have been implicated in progression of systemic lupus erythematosus (SLE). Additionally, CD1a-restricted autoreactive T cells have been implicated in psoriasis and the response to insect venoms. Since Group 1 CD1-restricted T cells recognizing mycobacterial lipids are expanded in patients infected with *Mycobacterium tuberculosis* or vaccinated with Bacillus Calmette-Guérin, it is likely that these cells exhibit adaptive-like properties and play a protective role during infection. A major challenge in studying the functions of Group 1 CD1 molecules is their absence from mice. Some researchers have therefore resorted to analyzing CD1-restricted T cell responses in guinea pigs, which express several CD1 isoforms. Guinea pigs induce Group 1 CD1-restricted T cell responses to mycobacterial lipids (15), suggesting adaptive-like functions of these cells. Other studies have employed a humanized CD1 transgenic mouse model (16) or humanized immunodeficient mice engrafted with human fetal thymic tissue and hematopoietic stem cells (17). These studies similarly revealed that *M. tuberculosis*-reactive Group 1 CD1-restricted T cells exhibit adaptive-like functions, suggesting promise for the development of lipid-based vaccines for mycobacterial and other infections. Studies with human CD1 transgenic mice also suggested a role of CD1b-restricted T cells in the generation of skin inflammation (18). Thus, as discussed in the review article by Lepore et al., the available evidence indicates that Group 1 CD1-restricted T cells share many functional properties with conventional, MHC class I-restricted T cells. Group 1 CD1-restricted T cells also hold promise for immunotherapy, as illustrated by studies showing that a subset of CD1c-restricted T cells exhibits strong reactivity for methyl-lysophosphatidic acid, a lipid antigen produced by leukemic cells (19). When adoptively transferred into immunodeficient mice, such T cells were able to limit the spread of CD1c-expressing human leukemia cells (19). Additionally, the CD1a-presented skin antigen, squalene (20), is a widely used adjuvant that enhances vaccine efficacy. Other antigens presented by Group 1 CD1 molecules could be similarly employed to modulate CD1-restricted T cell responses during vaccination or immunotherapy.

CD1d-restricted T cells are also called natural killer T (NKT) cells, which includes Type I or invariant NKT (iNKT) cells expressing semi-invariant TCRs, and Type II or diverse NKT (dNKT) cells expressing more diverse or oligoclonal TCRs (21). NKT cells have been targeted for immunotherapy of disease with ligands such as α-galactosylceramide (α-GalCer) for iNKT cells (22), or sulfatide for dNKT cells (23).

iNKT cells were originally identified as a subset of T cells expressing an invariant TCR in mice and humans (11, 24). The reactivity of these cells was subsequently shown to be restricted by CD1d (25). iNKT cells are highly conserved between mice, humans and other mammalian species (26). The iNKT TCR is made of Vα14-Jα18 and Vβ8.2/7/2 chains in mice or homologous Vα24-Jα18 and Vβ11 chains in humans (2, 3, 21). In mice, iNKT cells are most abundant in liver and adipose tissue, where they can represent up to 30–40% of the T cell population, are present at significant numbers in peripheral blood, spleen, thymus, and bone marrow (1–5% of all mature T cells), and are also found in lymph nodes, skin, and mucosal surfaces in the intestine and lungs (<1% of all T cells). However, these cells are quite rare and have variable frequency in humans (e.g., 0.005–0.2% of all T cells in peripheral blood). A variety of endogenous and exogenous lipid antigens for these cells have been identified (7, 8). All iNKT cells react with α-GalCer (27), an optimized version of a natural product originally isolated from a marine sponge, and α-GalCer-loaded CD1d tetramers selectively bind with iNKT cells. As described in the review by Ren et al., genetic tools to study iNKT cells include Jα18- and CD1d-deficient mice, as well as mouse Vα14-Jα18 and human Vα24-Jα18 TCR transgenic animals. iNKT cells express markers that are characteristic of the natural killer (NK) cell lineage, exhibit an activated or memory phenotype, are enriched in liver and mucosal tissues, have a tendency for autoreactivity, and are unable to generate classical memory responses. In addition to TCR-mediated activation signals, these cells are highly responsive to innate immune activation signals, such as innate cytokines produced in response to toll-like receptor (TLR) signaling in antigen-presenting cells (28). iNKT cells often become activated during situations of sterile inflammation involving release of damage-associated molecular patterns, also called alarmins. As discussed in the perspective by Ferhat et al., the alarmin IL-33 might play a key role in recruiting iNKT cells to the inflammatory site and in enhancing their regulatory and effector functions. iNKT cells can produce a variety of pro- and anti-inflammatory cytokines, and subsets of iNKT cells biased for production of type 1, 2, or 3 cytokines, called iNKT1, iNKT2 and iNKT17 cells, respectively, are enriched in different tissues (29). iNKT cells acquire these unusual properties during their development in the thymus, as discussed in the reviews by Krovi and Gapin, Wang and Hogquist, Yang et al., and Dashtsoodol et al.. A key driver in the acquisition of an innate effector program by these cells is the induction of the transcription factor promyelocytic leukemia zinc finger (PLZF) during thymic selection on CD1d-expressing CD4^+^CD8^+^ (double-positive) thymocytes (30, 31). Additionally, iNKT cells undergo unique epigenetic modifications in the thymus that influence their development, maturation and functional differentiation. Following the activation of iNKT cells by lipids and/or innate signals, these cells can activate and modulate a variety of innate and adaptive immune cells and thus impact overall immune responses and disease (32). The review article by Cortesi et al. discusses how reciprocal interactions between iNKT cells and mononuclear phagocytes in tissues impact physiological and pathological conditions. Additionally, the review articles by  Lang, Doherty et al., and Fujii et al. discuss how iNKT cells can interact with B cells to influence humoral immune responses, and Fujii et al. further review the capacity of iNKT cells to license dendritic cells to augment humoral and cellular immune responses.

Consistent with their potent immunomodulatory properties, iNKT cells contribute to protective immune responses against a variety of microorganisms, as discussed in the review articles by Kinjo et al., Trottein and Paget, and Huang et al.. For example, the original research article by Paquin-Proulx et al. provides evidence for dynamic regulation of iNKT cell numbers and functions during *M. tuberculosis* infection, suggesting a role for these cells in immunity against this organism. Interestingly, several microbial organisms, especially viruses that cause persistent infections, can evade CD1d-restricted immune responses (33), a topic discussed in the review article by Schönrich and Raftery. One particularly clever way to evade iNKT cell responses is illustrated by human immunodeficiency virus (HIV), which can infect iNKT cells, resulting in their depletion during disease progression (34). The original research article by Singh et al. provides evidence that patients with non-progressive HIV infection contain functionally competent iNKT cells, whereas anti-retroviral treatment of patients with progressive infection fails to restore iNKT cell functions.

iNKT cells have been implicated in natural immunity against tumors, a topic discussed by Terabe and Berzofsky and Stolk et al.. These cells also contribute to a variety of autoimmune and inflammatory diseases (28). The review by  Bagchi et al. focuses on the role of iNKT cells in autoimmune diseases such as SLE, psoriasis, diabetes, and rheumatoid arthritis that are characterized by dyslipidemia. iNKT cells also play a role in the generation of inflammatory bowel disease, as illustrated by the original research article by Lee et al., which provides evidence for a role of iNKT cells in suppressing disease in a model of colitis mediated by pathogenic CD8^+^ T cells. The review articles by Lezmi and Leite-de-Moraes, Iwamura and Nakayama, and Ryu et al. focus on the role of lung iNKT cells in asthma and other pulmonary disorders. These cells also contribute to the pathogenesis of alcoholic and non-alcoholic fatty liver disease, which is discussed in the review articles by Huang et al. and Marrero et al.. The review article by Ververs et al. describes the response of iNKT cells to metabolic activation in diseases such as atherosclerosis and obesity. Several review articles, including those by Park et al., Satoh and Iwabuchi, and  Ren et al., discuss the role of iNKT cells in the development of obesity and obesity-associated insulin-resistance, which is influenced by many different factors (35, 36). The divergent findings obtained in different laboratories for the role of iNKT cells in obesity-associated conditions might be caused in part by different models of iNKT cell-deficiency employed, a possibility discussed in the review article by Ren et al.. Additionally, differences in the microbiota present in different mouse colonies might play a role, as the functional status of iNKT cells is impacted by microbiota (37–39). The topic of functional alterations in iNKT cells imparted by gut microbiota is discussed by Marrero et al. in the context of liver diseases and by Mortier et al. in the context of rheumatic diseases. Additionally, the original research article by De Giorgi et al. provides evidence that the abundance of certain microbiota in the gut of diabetes-prone non-obese diabetic mice increases the intestinal iNKT17 subset in these animals, possibly contributing to disease pathogenesis.

The immunomodulatory properties of iNKT cells have been exploited for the development of disease prophylaxis and therapy (22). Many of these studies have been performed with the iNKT cell ligand α-GalCer or its structural analogs. The adjuvant activities of iNKT cells have been exploited for the development of vaccines against microbes and tumors, as discussed in the review articles by Lang, Doherty et al., Fujii et al., and  Kinjo et al.. As discussed by  Kinjo et al., glycolipid activation of iNKT cells during infection can enhance antimicrobial immunity and hasten microbial clearance. Many studies have explored the therapeutic activities of iNKT cells against tumors and, as discussed in the review articles by  Takami et al. and  King et al., multiple clinical trials have already been performed. The therapeutic potential of iNKT cells in autoimmune diseases is discussed by  Van Kaer and Wu.. In addition to these glycolipid antigen-mediated methods to elicit the therapeutic properties of iNKT cells, diseases where iNKT cells play a pathogenic role might benefit from iNKT cell depletion, a possibility that is being explored in sickle cell disease with antibodies directed against the invariant iNKT TCR (40).

dNKT cells express a more diverse TCR repertoire than iNKT cells (21, 23). In mice, dNKT cells are less prevalent than iNKT cells, whereas in humans dNKT cells outnumber iNKT cells. dNKT cells interact with multiple different lipid antigens, and a prominent subset reacts with the self-antigen sulfatide. While the sulfatide-reactive subset can be identified with sulfatide-loaded CD1d tetramers, rigorous identification of the entire dNKT cell population remains challenging. Curiously, as discussed in the review article by Nishioka et al., a subset of dNKT cells can react with hydrophobic peptides, including a peptide derived from type II collagen. As discussed in the review by Ren et al., the functions of dNKT cells are often deduced from studies comparing CD1d-deficient mice lacking both iNKT and dNKT cells with Jα18-deficient mice lacking only iNKT cells. dNKT cells share with iNKT cells expression of NK and memory markers, response to innate activation signals, innate-like effector functions, and absence of immunological memory. As discussed in the review article by Singh et al., dNKT cells can play both protective and pathogenic roles in a variety of diseases. Several review articles included in this Research Topic discuss the roles of dNKT cells in specific diseases. Trottein and Paget focus on lung infections, Terabe and Berzofsky and Stolk et al. on cancer, Iwamura and Nakayama on asthma, Satoh and Iwabuchi on obesity and insulin resistance, and Marrero et al. on inflammatory diseases of the liver and gut. In some conditions, such as cancer and steatohepatitis, dNKT appear to play opposing roles to iNKT cells (41). Emerging evidence, discussed in the review article by Marrero et al., has revealed substantial cross-regulation between iNKT and dNKT cells.

Sulfatide has been employed to target the sulfatide-reactive subset of dNKT cells for therapeutic purposes (23). As discussed in the review articles by Singh et al. and Marrero et al., sulfatide has beneficial effects in some infections and models of autoimmunity and liver inflammation. dNKT cells reactive with collagen type II peptide have also been targeted therapeutically (using cognate peptide), employing an experimental model of rheumatoid arthritis, as discussed in the review article by Nishioka et al..

The antigen-binding groove of MR1 is lined with aromatic residues, which facilitates binding with microbial vitamin B metabolites and similar compounds (42). The majority of MR1-restricted T cells express semi-invariant TCRs (43). Such cells are called mucosal-associated invariant T (MAIT) cells, were first identified as a clonally enriched T cell population in humans and mice (44, 45), and were subsequently shown to be restricted by MR1 (46) and to react with vitamin B metabolites (47). MAIT cells express Vα19-Jα33 chains in mice and homologous Vα7.2-Jα33 chains in humans, and these cells can be identified by staining with vitamin B metabolite-loaded MR1 tetramers. In humans, they represent ~3–5% of T cells in peripheral blood and intestine and up to 40% of T cells in liver. However, these cells are very rare in laboratory mice (<1% of all T cells). In addition to vitamin B metabolites, MAIT cells can react with a range of drugs and drug-like molecules, which can either activate or suppress these cells (48). Genetic tools to study MAIT cells include MR1- and Jα33-deficient mice and transgenic mice carrying the invariant Vα19-Jα33 TCR. As discussed in the review article by Garner et al., many of the studies on MAIT cells have been guided by the available biology on iNKT cells. MAIT cells share with iNKT cells an effector-memory phenotype, enrichment in liver and mucosal sites, expression of PLZF that drives an innate effector phenotype, response to innate signals, and the absence of classical immune memory. The antigen-specificity and activation requirements of MAIT cells are discussed in the review article by  Xiao and Cai. Emerging evidence, discussed in the review article from Dias et al., has revealed functional heterogeneity within the MAIT population with regard to cytokine production, akin to the iNKT1 and iNKT17 subsets of iNKT cells (49). MAIT cells have been implicated in immune responses against a variety of pathogens, including but not limited to organisms containing vitamin B metabolites, a topic discussed in the review article by  Trottein and Paget for lung infections. For example, the original research article by Paquin-Proulx et al. reports increased frequency of MAIT cells in individuals infected latently with *M. tuberculosis*, an organism that contains an intact riboflavin synthesis pathway, suggesting a potential role in disease pathogenesis. Moreover, MAIT cells can infiltrate tumors, and their potential role in anti-tumor immunity is discussed in the review article by Stolk et al.. The original research article by Solders et al. shows that MAIT cells are recruited to the intervillous space of the placenta, where they might play a role in fetal-maternal interactions. The review articles by Lezmi and Leite-de-Moraes and Iwamura and Nakayama discuss the potential role of MAIT cells in allergic asthma. The review article by Huang et al. discusses the role of these cells in inflammatory diseases of the liver, and the review article by Chiba et al. discusses their functions in animal models of autoimmune and inflammatory diseases. Finally, like iNKT cells, the development and functions of MAIT cells are substantially modulated by the presence of microbiota, which has a significant impact on disease, a topic discussed in the review article by Mortier et al. for rheumatic diseases. Although not yet fully explored, MAIT cells hold significant potential for therapeutic targeting, using vitamin B metabolites or drug-like molecules that could be employed to either potentiate or suppress MAIT cell responses.

Recent studies have identified subsets of MR1-restricted T cells that do not express the invariant MAIT TCR (42, 50). Some of these cells react with riboflavin-based antigens and exhibit innate-like effector functions, whereas others have a distinct antigen-reactivity and exhibit characteristics of adaptive immune effector functions, as discussed in the review article by Lepore et al.. Thus, these findings suggest additional similarities in the biology of MR1- and CD1d-restricted T cells.

The articles included in this Research Topic illustrate the breath of immune responses regulated by CD1- and MR1-restricted T cells, which include subsets with innate- and adaptive-like properties. An attractive property of the CD1 and MR1 antigen presentation systems, as compared with the classical MHC antigen presentation system, is that they can be more easily targeted in vaccines and immunotherapies. CD1 and MR1 exhibit limited polymorphism and are recognized by T cells that include subsets expressing conserved (i.e., public) TCRs (14). As such, it is feasible to target these cells across the genetically diverse human population in an antigen-specific manner. A better understanding of the basic biology of these cells and their role in different disease processes, together with the development of improved tools to target them, should facilitate the realization of this therapeutic promise.

## Author Contributions

KI and LV served as co-editors for the Research Topic and edited the manuscript. LV wrote the first draft.

### Conflict of Interest Statement

The authors declare that the research was conducted in the absence of any commercial or financial relationships that could be construed as a potential conflict of interest.
